# Recombinant Expression of *Serratia marcescens* Outer Membrane Phospholipase A (A1) in *Pichia pastoris* and Immobilization With Graphene Oxide-Based Fe_3_O_4_ Nanoparticles for Rapeseed Oil Degumming

**DOI:** 10.3389/fmicb.2019.00334

**Published:** 2019-02-21

**Authors:** Peizhou Yang, Suwei Jiang, Yun Wu, Zhigang Hou, Zhi Zheng, Lili Cao, Mingrui Du, Shaotong Jiang

**Affiliations:** Anhui Key Laboratory of Intensive Processing of Agricultural Products, College of Food and Biological Engineering, Hefei University of Technology, Hefei, China

**Keywords:** phospholipase, enzymatic degumming, immobilization, graphene oxide, magnetic nanoparticle, *Pichia pastoris*

## Abstract

Enzymatic degumming is an effective approach to produce nutritional, safe, and healthy refined oil. However, the high cost and low efficiency of phospholipase limit the application of enzymatic degumming. In this study, an 879 bp outer membrane phospholipase A (A1) (OM-PLA1) gene encoding 292 amino acid residues was isolated from the genome of *Serratia marcescens*. The recombinant OM-PLA1 profile of appropriately 33 KDa was expressed by the engineered *Pichia pastoris* GS115. The OM-PLA1 activity was 21.2 U/mL with the induction of 1 mM methanol for 72 h. The expression efficiencies of OM-PLA1 were 0.29 U/mL/h and 1.06 U/mL/OD600. A complex of magnetic graphene oxide (MGO) and OM-PLA1 (MGO-OM-PLA1) was prepared by immobilizing OM-PLA1 with graphene oxide-based Fe_3_O_4_ nanoparticles by cross-linking with glutaraldehyde. The content of phosphorus decreased to 5.1 mg/kg rapeseed oil from 55.6 mg/kg rapeseed oil with 0.02% MGO-OM-PLA1 (w/w) at 50°C for 4 h. MGO-OM-PLA1 retained 51.7% of the initial activity after 13 times of continuous recycling for the enzymatic degumming of rapeseed oil. This study provided an effective approach for the enzymatic degumming of crude vegetable oil by developing a novel phospholipase and improving the degumming technology.

## Introduction

High-quality edible oils should meet the requirements of good stability, long shelf life, bland odor and taste, good nutritional quality, abundant vitamins, and no contaminants (Nykter et al., 2006; Goyal et al., 2014). Most types of crude vegetable oils extracted using traditional pressing or extraction technology contain colloidal substances, mainly phospholipids (Gofferje et al., 2014). Phospholipids are usually combined with impurities, such as proteins, mucus, and trace metals, in crude vegetable oils (Elena et al., 2016). These colloidal substances directly reduce the quality of oils and affect subsequent refining processes (Elena et al., 2017). Water degumming removes 88.5% and retains 51.1 ppm of phosphorus in oil (Zufarov et al., 2009). The residual phosphorus in hydrated oil could be removed by using acid and enzymatic degumming approaches. Enzymatic oil degumming was industrially proven and well accepted for its advantages of energy saving and environmental protection compared with acid degumming (Sampaio et al., 2015). However, the current high cost of phospholipase increases the production cost of oil and limits the wide application of enzymatic degumming. In addition, the residual phospholipase cannot be recovered and reused and still remain in the oil by free phospholipase degumming. Therefore, the preparation of high-quality phospholipase and its reuse by immobilization determine the application degree of enzymatic degumming (Sampaio et al., 2015).

Outer-membrane phospholipase A (A1) (OM-PLA1) is an enzyme present in the outer membrane of Gram-negative bacteria (Dekker, 2000). OM-PLA1 hydrolyzes the acylester bonds in phospholipids and lysophospholipids. OM-PLA1 possesses a more conserved and specific determinant in the lipid headgroup compared with other phospholipases (Martin et al., 2000). In the glyceryl complexes, OM-PLA1 has high tolerance to the headgroups of triglyceride, phosphatidylglycerol, phosphatidylcholine, and phosphatidylethanolamine (Stanley et al., 2007). In addition, crude vegetable oils widely contain the glyceryl complexes of glycerophosphate, glycerophosphatidyl choline, and glycerophosphatidyl ethanolamine (Sampaio et al., 2015). OM-PLA1 is an effective alternative for the degumming of crude plant oil (Manjula et al., 2010). Therefore, in this study, a novel *OM-PLA1* from *S. marcescens* was applied to crude rapeseed oil degumming.

The protein loading of magnetic graphene oxide (MGO) is much higher than that of granular activated carbon, diatomite and powdered activated carbon (Deng et al., 2013). Graphene oxide is an important derivative of graphene. The surface of graphene oxide has many oxygen-containing functional groups (hydroxyl, epoxy, carboxyl, carbonyl) and can be loaded with metal or metal oxides (Deng et al., 2008). The surface of graphene oxide Fe_3_O_4_ nanocomposite is adhering to magnetic nanoiron with superparamagnetism, which makes it have good magnetic separation characteristics and strong adsorption capacity. In addition, graphene oxide Fe_3_O_4_ nanocomposite also has advantages of high specific surface area, super strength, high activity recovery, and immobilization efficiency of enzyme compared to other immobilization approaches (Huang et al., 2014; Xie and Huang, 2018).

**FIGURE 1:**
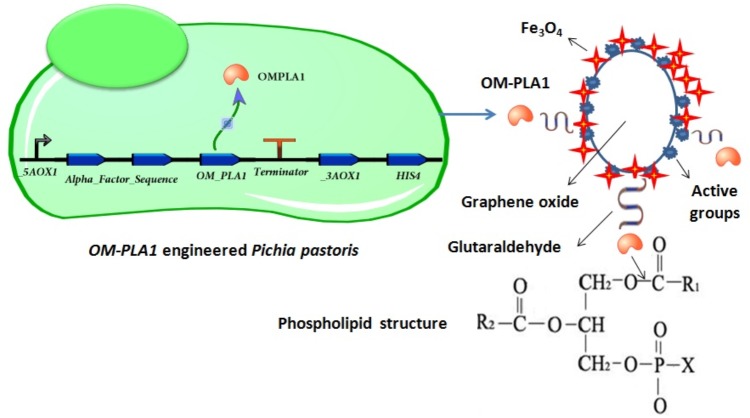
Technical path of this study.

As an edible vegetable oil, crude rapeseed oil contains substantial phospholipids and causes oil discoloration and low oil quality (Yang et al., 2013; Jiang X. et al., 2014). Engineered *P. pastoris* can effectively express exogenous protein (Tolner et al., 2006). In this study, the *S. marcescens OM-PLA1* expression vector was transformed into the cells of *P. pastoris* GS115. The gene expression and OM-PLA1 immobilization for crude rapeseed oil degumming were investigated to develop an effective approach for the enzymatic degumming of vegetable oil (Figure 1).

## Materials and Methods

### Materials

*S. marcescens* isolated from the intestine of smelly mandarin fish (*Siniperca chuatsi*) was preserved in College of Food and Biological Engineering, Hefei University of Technology. The smelly mandarin fish is a processed food product sold in market using traditional fermentation technology (Yang et al., 2017). The use of the dead mandarin fish is not unnecessary to obtain the approval from the institutional review board or ethics committee prior to commencing this study. *P. pastoris* GS115 and plasmid pPIC9K were provided by Dr. Huang from Qingdao Vland Biotech Company. Chemical reagents were from Beijing Transgen Biotech Company. Gene sequencing and primer synthesis were performed by Shanghai Sangon Biotech.

### *OM-PLA1* Cloning and Engineered *P. pastoris* Construction

According to the gene sequences of *OM-PLA1* in the NCBI database (HG326223.1), primer pair Us-OM-PLA1 and Ds-OM-PLA1 was synthesized to amplify the *S. marcescens* genome for *OM-PLA1* cloning using PCR amplification instrument (TOMOS, United States) (Table 1). A phylogenetic tree was drawn by using Lagergene MegAlign software. The *OM-PLA1* gene was inserted into plasmid pEASY-E1. The recombinant plasmid was named pEASY-OM-PLA1 after sequencing confirmation. Then, pEASY-OM-PLA1 was used to amplify OM-PLA1 with upstream primer Us-*Snab*I-OM-PLA1 and downstream primer Ds-*Avr*II-OM-PLA1. OM-PLA1 carrying cohesive ends was inserted into the pPIC9K plasmid digested by *Snab*I and *Avr*II (Gohel and Singh, 2012; Gamerith et al., 2017). The recombinant plasmid was named pPIC9K-OM-PLA1 by sequencing confirmation. pPIC9K-OM-PLA1 was transformed into *P. pastoris* GS115 by electroporation (Guo et al., 2015). The electrophoresis device and gel imaging system for DNA test were from Bio-Rad Company (United States).

### OM-PLA1 Inducible Expression and Purification

The inducible expression of OM-PLA1-engineered *P. pastoris* GS115 was investigated under different methanol concentrations and induction times (Bredell et al., 2018). Fermentation media were prepared by yeast extract 1% (w/v), peptone 2% (w/v), 100 mM pH6 potassium phosphate buffer, YNB 1.34% (w/v), biotin 4 × 10^-5^ % (w/v), and glycerol 1% (w/v). The engineered *P. pastoris* colony was cultured in fermentation media at a shaking speed of 200 rpm at 30°C for 36 h. The collected *P. pastoris* cells were inoculated into the fermentation media added with 1 mM methanol. The final cell concentration at OD600 of 20 was cultured with a shaking speed of 200 rpm at 30°C. A Ni^2+^-chelating affinity chromatography column (Amersham Pharmacia Biotech, United Kingdom) was pre-equilibrated by a pH 8 buffer prepared by 20 mM imidazole, 50 mM NaH_2_PO_4_, and 300 mM NaCl. After the recombinant OM-PLA1 was purified by the column (Wang et al., 2009), OM-PLA1 profile analysis was performed by SDS-PAGE (Bio-Rad, United States) (Bredell et al., 2018).

**Table 1:** Primers for gene cloning and vector construction.

Primer name	Sequences
Us-OM-PLA1	5′-TATGCGCATTTTGTCAGGGA-3′
Ds-OM-PLA1	5′-GATTACATAATATCGTTCAGC-3′
Us-*Snab*I- OM-PLA1	5′-GGGAAA**TACGTA**TATGCGCATTTTGTCAGGGA-3′
Ds-*Avr*II- OM-PLA1	5′-GGGAAA**CCTAGG**GATTACATAATATCGTTCAGC-3′

### Fe_3_O_4_/Graphene Oxide Preparation and OM-PLA1 Immobilization

Magnetic Fe_3_O_4_/graphene oxide (MGO) was prepared by mixing 1.5 g of freeze-dried graphene oxide, 0.3 g of FeSO_4_⋅7H_2_O, and 0.4 g of FeCl_3_⋅6H_2_O in 50 mL of deionized water (Hasanzadeh and Shadjou, 2013; Amirbandeh and Taheri-Kafrani, 2016; Zhuang et al., 2016). OM-PLA1 was immobilized by mixing glutaraldehyde, OM-PLA1, 1 g of MGO, and 50 mL of the immobilization buffer prepared by 0.01 M citric acid and 0.02 M hydrogen phosphate disodium. The parameters of pH, treatment time, and glutaraldehyde concentration were investigated to produce the MGO-OM-PLA1 complex. MGO-OM-PLA1 was separated and recovered by magnets and washed three times by the immobilization buffer (Qiu et al., 2010). Field emission scanning electron microscope (FE-SEM, Hitachi SU8020, Japan) and Fourier transform infrared spectroscope (FTIR, Thermo Nicolet 67, United States) were used to observe the microstructure and the spectral characteristic of MGO-OM-PLA1, respectively (He et al., 2013; Huang et al., 2015).

**FIGURE 2:**
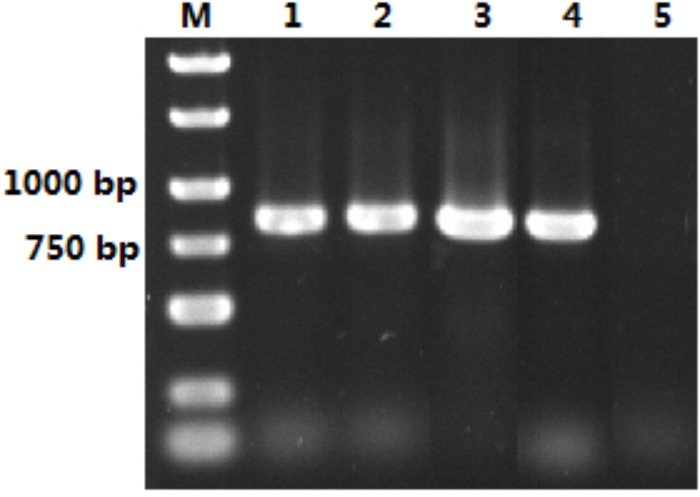
Isolation of *OM-PLA1* from the genome of *S. marcescens.* Note: Lane M represented DNA Marker; lane 1–4 represented PCR amplification product of *OM-PLA1*; lane 5 represented the control.

**FIGURE 3:**
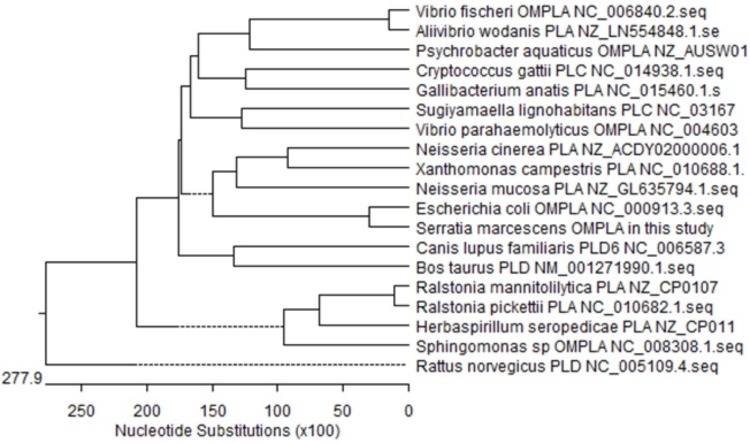
Phylogenetic tree of phospholipase genes from 19 different microorganisms.

### MGO-OM-PLA1 Degumming of Rapeseed Oil

Enzymatic degumming was performed by adding MGO-OM-PLA1 in the crude rapeseed oil (Jiang X. F. et al., 2014). The phosphorus content of crude rapeseed oil was 55.6 mg/kg. Enzymatic degumming was carried out with the addition of 0.02% MGO-OM-PLA1 (w/w) into a weight of 150 g crude rapeseed oil at 50°C. MGO-OM-PLA1 was recovered by magnets to terminate the degumming reaction. The times of rapeseed oil degumming were determined when the phosphorus concentration was lower than 10 mg/kg rapeseed oil at each time. The recovered MGO-OM-PLA1 particles were reused for the next degumming of rapeseed oil.

**FIGURE 4:**
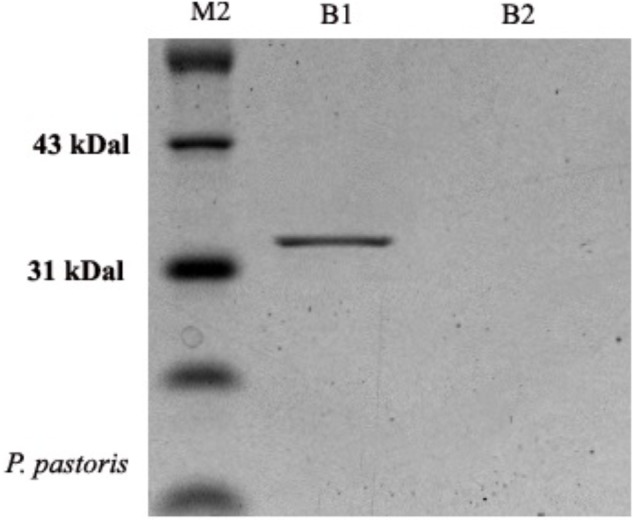
Profile of recombinant OM-PLA1 from *P. pastoris* by SDS-PAGE approach. Note: Lane M2 represented protein marker; B1 and B2 respectively represented the protein from the engineered and wild type *P. pastoris* GS 115.

**FIGURE 5:**
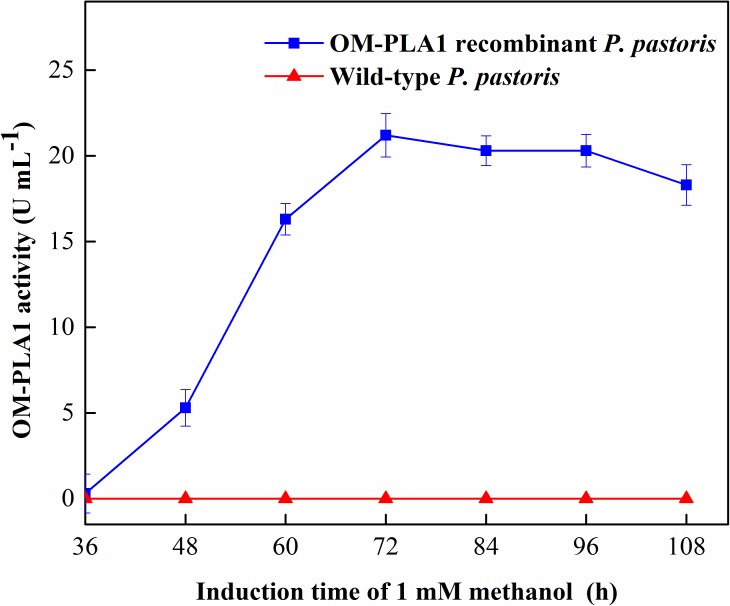
Effect of induction time of 1 mM methanol on the expression of recombinant OM-PLA1.

### Determination of Phosphorus Content and OM-PLA1 Activity

Phosphorus content was determined by a spectrophotometric method in accordance with Official Method Ca 12–55 using ultraviolet visible near-infrared spectrophotometer (Agilent CARY5000, United States) (Chen et al., 2014). The activity of OM-PLA1 was measured by the colorimetric assay using a pH indicator (Araújo and Radvanyi, 1987; Pires et al., 2017). Triton–soybean lecithin was prepared by using 10 g of soybean lecithin dissolved in 200 mL of 0.02% Triton X-100 solution (w/v). A 98 mL volume of Triton–soybean lecithin and 2 mL of fermentation solution were mixed. The pH of the mixture was adjusted to 10 by adding 0.01 M NaOH. The activity of OM-PLA1 was determined based on the consumed volume of NaOH. The activity of phospholipase is defined as a unit of phospholipase activity required to hydrolyze phospholipids for 1 min to produce 1 μmol of free fatty acids (Sutto-Ortiz et al., 2017). Data are presented as mean ± standard deviation (SD). Figures were drawn using Software Origin.

**FIGURE 6:**
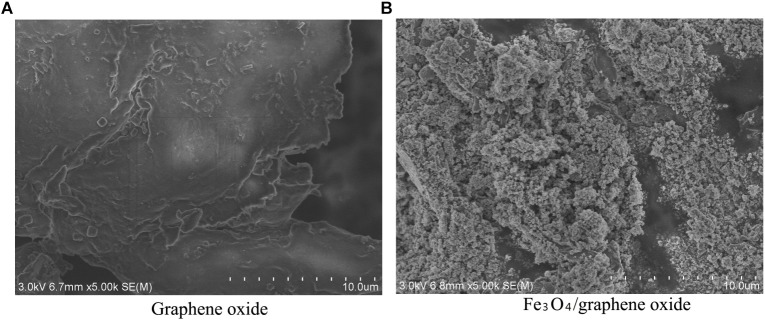
Microcosmic difference between **(A)** GO and **(B)** prepared Fe_3_O_4_/GO.

## Results

### *S. marcescens OM-PLA1* Cloning and Phylogenetic Tree Analysis

The size of the isolated *S. marcescens OM-PLA1* is 879 bp after sequencing confirmation (Figure 2), which encodes 292 amino acid residues. The phylogenetic tree of phospholipase sequences from 19 different species was drawn (Figure 3). *S. marcescens* OM-PLA1 showed the closest relationship to *Escherichia coli OM-PLA1* NC_000913.3 among the tested phospholipase-producing microorganisms. A close genetic relationship also existed between *Vibrio parahaemolyticus* OM-PLA NC_004603.1 and *Aliivibrio wodanis* PLA NZ_LN554848.1, *Ralstonia mannitolilytica* PLA NZ_CP010799.1, and *Ralstonia pickettii* PLA NC_010682.1. However, *S. marcescens* OM-PLA1 has a far genetic relationship with *Sphingomonas* sp. OM-PLA NC_008308.1 compared with other phospholipase genes.

### Recombinant Expression of OM-PLA1 in *P. pastoris*

The molecular weight of OM-PLA1 was appropriately 33 KDa by SDS-PAGE (Figure 4). The induction time of methanol affecting the expression of recombinant OM-PLA1 was investigated (Figure 5). The wild-type *P. pastoris* could not produce OM-PLA1 during the induction processing of methanol. The engineered *P. pastoris* could effectively produce the recombinant OM-PLA1. The activity of OM-PLA1 was 21.2 U/mL after induction by 1 mM methanol for 72 h. The production efficiencies of treatment time and cell concentration were 0.29 U/mL/h and 1.06 U/mL/OD600, respectively.

**FIGURE 7:**
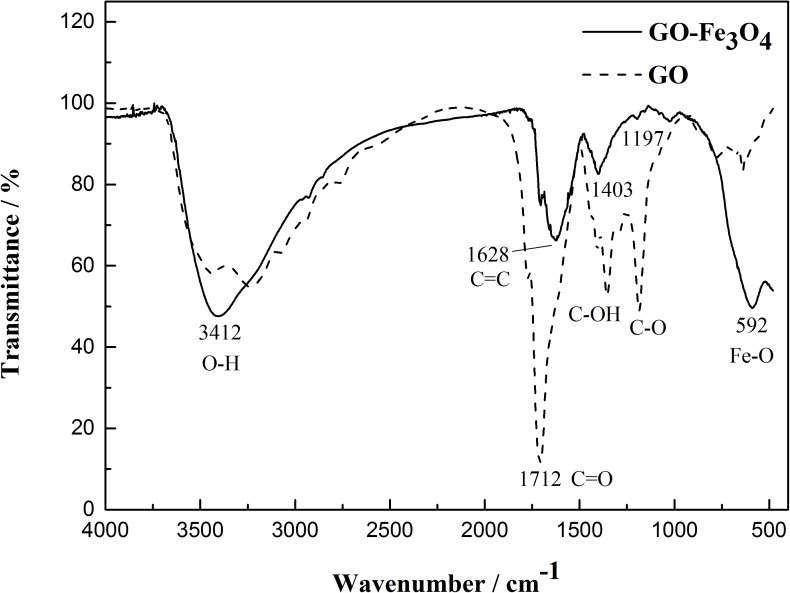
FTIR observation of graphene oxide-based Fe_3_O_4_ nanoparticles.

### FE-SEM Observation of the Fe_3_O_4_/GO Complex

FE-SEM was used to investigate the microcosmic difference between GO and the prepared Fe_3_O_4_/GO (Figure 6). The whole structure of GO was complete. The edge outline was clear and neat. The exfoliated lamellae of GO showed a smooth, thin, and large surface. No visible objects attached onto the surface of GO (Figure 6A). Figure 6B shows that the surface of the whole material was rough and convex. The iron oxide nanoparticles were largely anchored on the lamellae of GO in a highly dispersed state.

**FIGURE 8:**
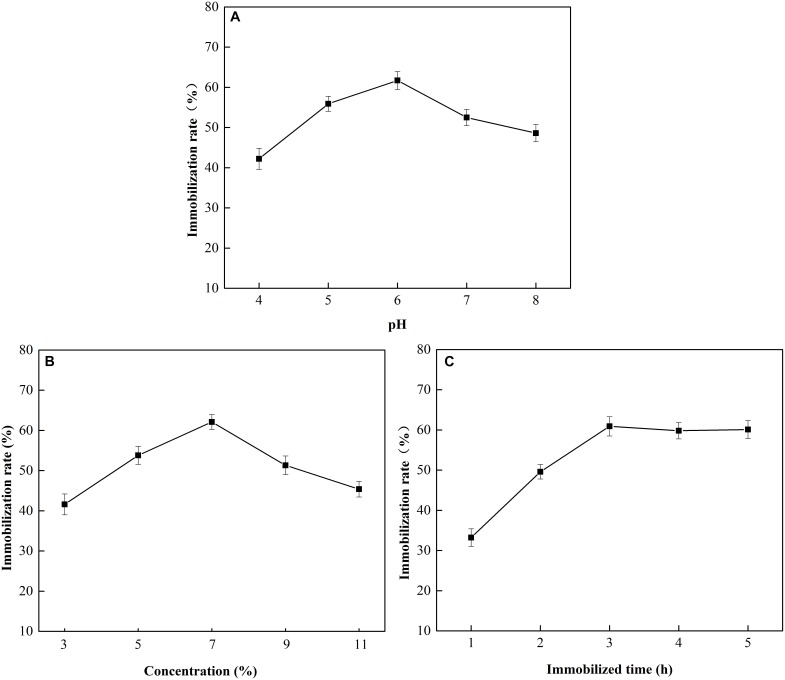
Effect of pH, glutaraldehyde concentration, and immobilization time on MGO-OM-PLA1 preparation. **(A–C)** Represented pH, glutaraldehyde concentration, and immobilization time, respectively.

**FIGURE 9:**
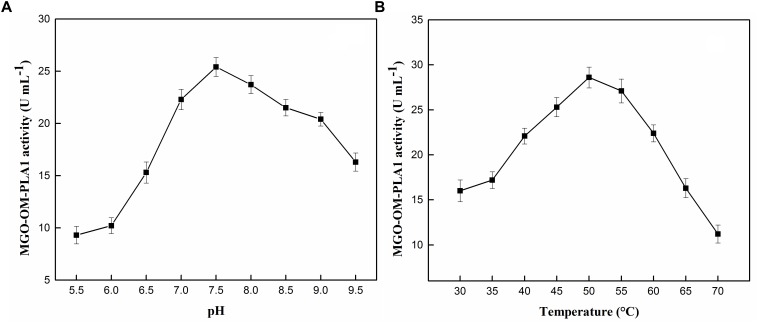
Effect of pH **(A)** and temperature **(B)** on the activity of MGO-OM-PLA1.

Very few of the GO were still exposed without attachment of granular objects. Fe_3_O_4_ particles showed spherical agglomeration. The thickness of coverage by Fe_3_O_4_ particles was inhomogeneous. The Fe_3_O_4_ particle size ranged from 10 to 30 nm. A small amount of Fe_3_O_4_ particles exceeded 50 nm of size.

### FTIR Spectral Analysis of Graphene Oxide-Based Fe_3_O_4_ Nanoparticles

FTIR spectroscopy was used to observe the preparation of MGO particles (Figure 7). Multiple oxygen-containing functional groups existed on the surface and edge of GO. The absorption peaks of MGO particles obviously differed from those of graphite oxide. The characteristic absorption peaks at 3412, 1712, 1628, 1197, and 592 cm^-1^ corresponded to the stretching vibrations of O-H, C = O, C = C, C-O, and Fe-O. In the Fe_3_O_4_/GO composite, the absorption peaks at 1712, 1628, and 1197 cm^-1^ were greatly weakened in comparison with those of GO. The absorption peak of epoxy at 1197 cm^-1^ almost disappeared in the Fe_3_O_4_-GO composite, which reflected that chemical bonding existed between these oxygen-containing functional groups and Fe_3_O_4_ particles. In addition, the Fe-O absorption peaks from Fe_3_O_4_-GO at 592 cm^-1^ were significantly strengthened, which further proved that a close combination existed between Fe_3_O_4_ and GO.

**FIGURE 10:**
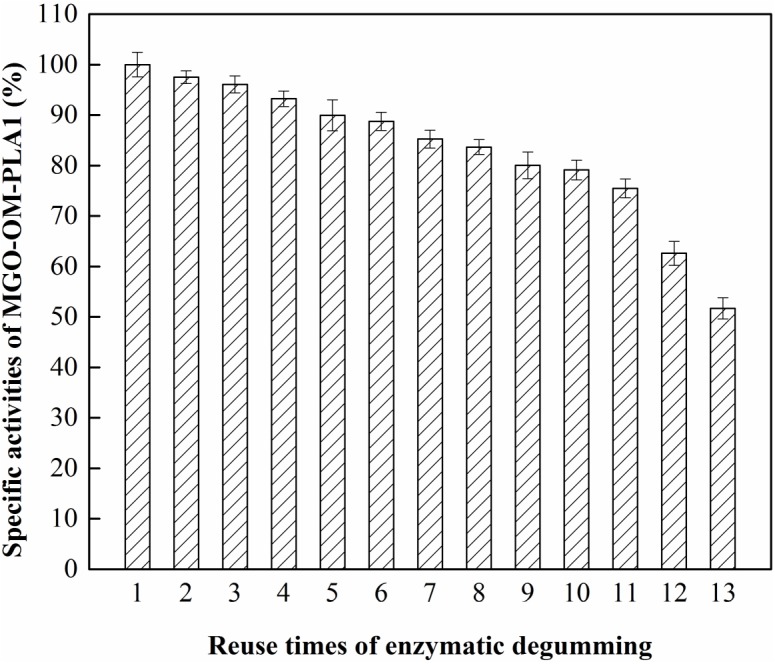
MGO-OM-PLA1 enzymatic degumming of crude rapeseed oil under the conditions of pH 7.5 at 50°C.

**FIGURE 11:**
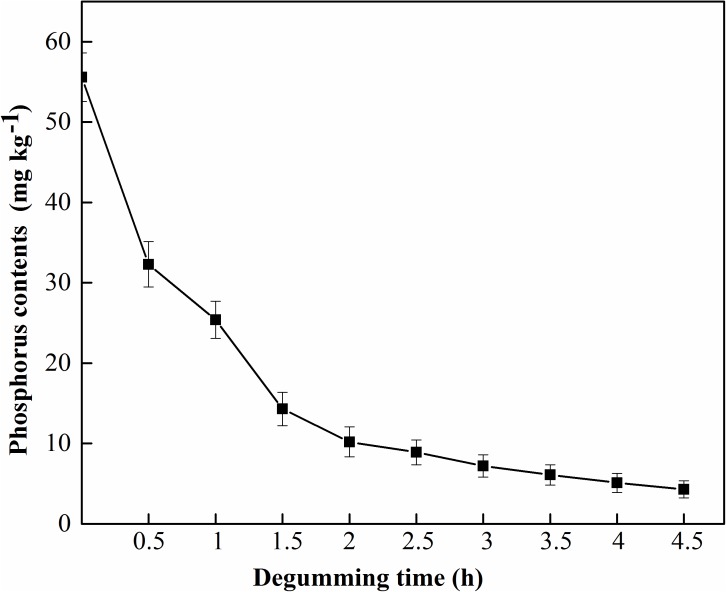
Effect of recycling times on MGO-OM-PLA1 activity under the conditions of pH 7.5 at 50°C.

**Table 2:** Expression of the phospholipase gene in recombinant host strains.

Phospholipase	Host strain	Expression efficiency
*S. marcescens* OM-PLA1	*P. pastoris*	21.2 U/mL, 0.29 U/mL/h, this study
*Fusarium oxysporum* PLB	*P. pastoris*	6.6 g/L (Su et al., 2017)
*Bacillus cereus* PLB	*P. pastoris*	4.5 g/L (Elena et al., 2016)
*Streptomyces violaceoruber* PLA2	*P. pastoris*	34.7 U/mL (Liu et al., 2015)
*Thermotoga lettingae* PLB	*E. coli*	Half-life of 240 min at 90°C (Wei et al., 2015)
*Pseudomonas fluorescens* PLB	*E. coli*	20.1% higher than the wild-type (Jiang et al., 2012)
*Bacillus cereus* PLC	*C. glutamicum*	5.5 g/L (Ravasi et al., 2015)

### MGO-OM-PLA1 Preparation

The effects of pH, glutaraldehyde concentration, and immobilization time on MGO-OM-PLA1 preparation were investigated (Figure 8). At pH 6, the immobilization efficiency of OM-PLA1 reached 61.7% (w/w), which was the highest among the set parameters (Figure 8A). The highest immobilization efficiency was achieved when the glutaraldehyde concentration (v/v) was 7% (Figure 8B). In addition, the efficiency of OM-PLA1 immobilization reached 60.9% (w/w) after 3 h of treatment (Figure 8C), which was the higher than those at other times.

### Rapeseed Oil Degumming

The effect of pH and temperature on the activity of MGO-OM-PLA1 was investigated (Figure 9A,B). Under the conditions of pH 7.5 (Figure 9A) and 50°C (Figure 9B), the activity of MGO-OM-PLA1 reached the highest among the set parameters. Therefore, in this study, the parameters of pH 7.5 and 50°C were used to investigate the enzymatic degumming of crude vegetable oils.

MGO-OM-PLA1 was used in the enzymatic degumming of crude rapeseed oil (Figure 10). The contents of phosphorus in crude rapeseed oil gradually decreased with prolonged treatment time. The content of 0.02% MGO-OM-PLA1 (w/w) was used to perform enzymatic degumming with pH 7.5 at 50°C. After degumming for 2.5 h, the phosphorus content was 8.9 mg/kg rapeseed oil (below 10 mg/kg) from the initial concentration of 55.6 mg/kg rapeseed oil. After 4 h of degumming, the phosphorus contents further decreased to 5.1 mg/kg rapeseed oil.

### Recovery and Reuse of MGO-OM-PLA1

The effect of recycling times on MGO-OM-PLA1 activity was investigated (Figure 11). The activity of MGO-OM-PLA1 gradually decreased with prolonged degumming time. MGO-OM-PLA1 retained 90% of the initial activity after degumming for five times. After 13 times of recycling, the activity of MGO-OM-PLA1 decreased to 51.7% of the initial activity of the enzyme. Therefore, the half life of MGO-OM-PLA1 activity was regarded as 13 times for the enzymatic degumming of rapeseed oil.

## DISCUSSION

The increase in oil yield and effective application on crude oils determines the development of enzymatic degumming. Enzymatic degumming has been applied in more than 30 crushing-refining plants. In general, a phosphorus content of below 10 mg/kg oil meets the actual requirement (Lamas et al., 2016). Previous reports on crude oil degumming mainly focused on the breeding of strains producing phospholipase A2, B, and C (Table 2). The phospholipases of the recombinant strains possess high application values in enzymatic degumming (Wei et al., 2015; Elena et al., 2017; Su et al., 2017). In this study, *S. marcescens* OM-PLA1 was effectively expressed in engineered *P. pastoris*. The activity and expression efficiency of OM-PLA1 were 21.2 U/mL and 0.29 U/mL/h, respectively. The immobilization product MGO-OM-PLA1 possessed excellent degumming capability of oil. The MGO-OM-PLA1 activity still retained 75 and 50% of the initial activity with 11 and 13 recycling times, respectively. In comparison with the reported 70% of the initial activity retained after seven recycles (Zhang et al., 2007), MGO-OM-PLA1 has the advantages of more application frequencies and higher degumming efficiency. Therefore, *S. marcescens* OM-PLA1 expressed by recombinant *P. pastoris* exhibited a potential application value in the enzymatic degumming of oil.

The material of immobilization is also a critical factor influencing the degumming of phospholipase. Octyl agarose, gelatin hydrogel, and cellulose triacetate have been applied to immobilize the commercial enzyme Lecitase (R) Ultra for oil degumming (Fernandez-Lorente et al., 2007; Sheelu et al., 2008; da Silva et al., 2017). In addition, sodium alginate and chitosan microparticles were crosslinked with Fe_3_O_4_ to immobilize phospholipase (Qu et al., 2016). In this study, graphene oxide was used to immobilize phospholipase OM-PLA1 for its advantage of a large number of functional groups on its surface, such as carboxyl, hydroxyl and epoxy groups, which allow easy reaction with compounds (Li et al., 2015). As a scaffold for enzyme immobilization, graphene oxide-based Fe_3_O_4_ nanoparticles possess a magnetic property (Shao et al., 2015), which facilitate easy recovery and reuse.

In this study, graphene oxide-based Fe_3_O_4_ nanoparticles were used to immobilize *S. marcescens* OM-PLA1 for the enzymatic degumming of rapeseed oil. The contents of phosphorus decreased to 8.9 mg/kg rapeseed oil from 55.6 mg/kg rapeseed oil with 2.5 h of degumming. Therefore, this study provided an effective approach for the enzymatic degumming of crude vegetable oil. However, some problems remain to be solved. First, the immobilization efficiencies of OM-PLA1 and graphene oxide-based Fe_3_O_4_ nanoparticles can still be increased. Second, the effect of the crosslinking agent glutaraldehyde on the activity of OM-PLA1 need further analysis. Third, the degumming effect of MGO-OM-PLA1 on other crude vegetable oils should be further explored.

## Conclusion

In this study, *S. marcescens OM-PLA1* gene with a size of 879 bp was isolated and expressed in the recombinant *P. pastoris* GS 115. The size of OM-PLA1 was appropriately 33 KDa. After 72 h of inducible time, the highest activity of OM-PLA1 was reached (21.2 U/mL). The production efficiencies of treatment time and cell concentration of OM-PLA1 were 0.29 U/mL/h and 1.06 U/mL/OD600, respectively. After 4 h of degumming, the phosphorus contents reached 5.1 mg/kg from 55.6 mg/kg using 0.02% (w/w) MGO-OM-PLA1 prepared by graphene oxide-based Fe_3_O_4_ nanoparticles and OM-PLA1. The half life of MGO-OM-PLA1 activity was 13 times for the enzymatic degumming of rapeseed oils with the initial phosphorus content of 55.6 mg/kg. Therefore, *S. marcescens OM-PLA1* gene could effectively express in the recombinant *P. pastoris*. In addition, the cross-linked copolymer MGO-OM-PLA1 possessed good stability and effective degumming of vegetable oils. This study has an important application value in the enzymatic degumming of crude vegetable oil.

## Author Contributions

PY designed the experiment scheme. SuJ wrote the manuscript. YW performed the experiments. ZH analyzed the data. ZZ drew the figures. LC measured the enzyme activity. MD cloned the OMPLA1 gene. ShJ proposed the idea.

## Conflict of Interest Statement

The authors declare that the research was conducted in the absence of any commercial or financial relationships that could be construed as a potential conflict of interest.

## References

[B1] Amirbandeh M. Taheri-Kafrani A. (2016). Immobilization of glucoamylase on triazine-functionalized Fe3O4/graphene oxide nanocomposite: improved stability and reusability. *Int. J. Biol. Macromol.* 93 1183–1191. 10.1016/j.ijbiomac.2016.09.092 27693337

[B2] Araújo A. Radvanyi F. (1987). Determination of phospholipase A2 activity by a colorimetric assay using a pH indicator. *Toxicon* 25 1181–1188. 10.1016/0041-0101(87)90136-X 3433293

[B3] Bredell H. Smith J. J. Gorgens J. F. van Zyl W. H. (2018). Expression of unique chimeric human papilloma virus type 16 (HPV-16) L1-L2 proteins in *Pichia pastoris* and *Hansenula polymorpha*. *Yeast* 35 519–529. 10.1002/yea.3318 29709079

[B4] Chen B. Xiao X. Li R. Zhao W. L. Yang K. D. Chen G. L. (2014). An improved method for determining the phosphorus content in vegetable oils. *Eur. Lipid J. Sci. Technol.* 116 548–552. 10.1002/ejlt.201300378

[B5] da Silva F. B. de Morais W. G. da Silva C. V. Vieira A. T. Batista A. de Faria A. M. (2017). Preparation and characterization of cellulose triacetate as support for lecitase ultra immobilization. *Molecules* 22:E1930. 10.3390/molecules22111930 29144385 PMC6150194

[B6] Dekker N. (2000). Outer-membrane phospholipase A: known structure, unknown biological function. *Mol. Microbiol.* 35 711–717. 10.1046/j.1365-2958.2000.01775.x 10692149

[B7] Deng J. H. Zhang X. R. Zeng G. M. Gong J. L. Niu Q. Y. Liang J. (2013). Simultaneous removal of Cd(II) and ionic dyes from aqueous solution using magnetic graphene oxide nanocomposite as an adsorbent. *Chem. Eng. J.* 226 189–200. 10.1016/j.cej.2013.04.045

[B8] Deng Y. Qi D. Deng C. Zhang X. Zhao D. (2008). Superparamagnetic high-magnetization microspheres with an Fe3O4@SiO2 core and perpendicularly aligned mesoporous SiO2 shell for removal of microcystins. *J. Am. Chem. Soc.* 130 28–29. 10.1021/ja0777584 18076180

[B9] Elena C. Cerminati S. Ravasi P. Rasia R. Peiru S. Menzella H. G. (2017). *B-cereus* phospholipase C engineering for efficient degumming of vegetable oil. *Process Biochem.* 54 67–72. 10.1016/j.procbio.2017.01.011

[B10] Elena C. Ravasi P. Cerminati S. Peiru S. Castelli M. E. Menzella H. G. (2016). *Pichia pastoris* engineering for the production of a modified phospholipase C. *Process Biochem.* 51 1935–1944. 10.1016/j.procbio.2016.08.022

[B11] Fernandez-Lorente G. Palomo J. M. Guisan J. M. Fernandez-Lafuente R. (2007). Effect of the immobilization protocol in the activity, stability, and enantioslectivity of Lecitase(R) Ultra. *J. Mol. Catal. B Enzym.* 47 99–104. 10.1016/j.molcatb.2007.04.008

[B12] Gamerith C. Vastano M. Ghorbanpour S. M. Zitzenbacher S. Ribitsch D. Zumstein M. T. (2017). Enzymatic degradation of aromatic and aliphatic polyesters by pastoris, P., expressed cutinase 1 from *Thermobifida cellulosilytica*. *Front. Microbiol.* 8:938. 10.3389/fmicb.2017.00938 28596765 PMC5443175

[B13] Gofferje G. Motulewicz J. Stabler A. Herfellner T. Schweiggert-Weisz U. Floter E. (2014). Enzymatic degumming of crude jatropha oil: evaluation of impact factors on the removal of phospholipids. *J. Am. Oil Chem. Soc.* 91 2135–2141. 10.1007/s11746-014-2559-2

[B14] Gohel S. D. Singh S. P. (2012). Cloning and expression of alkaline protease genes from two salt-tolerant alkaliphilic actinomycetes in *E. coli*. *Int. J. Biol. Macromol.* 50 664–671. 10.1016/j.ijbiomac.2012.01.039 22327111

[B15] Goyal A. Sharma V. Upadhyay N. Gill S. Sihag M. (2014). Flax and flaxseed oil: an ancient medicine & modern functional food. *J. Food Sci. Technol.* 51 1633–1653. 10.1007/s13197-013-1247-9 25190822 PMC4152533

[B16] Guo D. Tian H. Xu Y. Zheng S. (2015). Cloning and expression of β-Glucosidase from *Cassava* in *Pichia pastoris* GS115. *Adv. Appl. Biotechnol.* 333 11–20. 10.1007/978-3-662-46318-5_2

[B17] Hasanzadeh M. Shadjou N. (2013). (*Fe*3O4)-graphene oxide-SO3H as a new magnetic nanocatalyst for electro-oxidation and determination of selected parabens. *J. Nanosci. Nanotechnol.* 13 4909–4916. 10.1166/jnn.2013.7605 23901510

[B18] He G. Y. Liu W. F. Sun X. Q. Chen Q. Wang X. Chen H. Q. (2013). Fe3O4@graphene oxide composite: a magnetically separable and efficient catalyst for the reduction of nitroarenes. *Mater. Res. Bull.* 48 1885–1890. 10.1016/j.materresbull.2013.01.038

[B19] Huang J. Chang Q. Ding Y. B. Han X. Y. Tang H. Q. (2014). Catalytic oxidative removal of 2,4-dichlorophenol by simultaneous use of horseradish peroxidase and graphene oxide/Fe3O4 as catalyst. *Chem. Eng. J.* 254 434–442. 10.1016/j.cej.2014.05.136

[B20] Huang Y. H. Wang Y. Z. Pan Q. Wang Y. Ding X. Q. Xu K. J. (2015). Magnetic graphene oxide modified with choline chloride-based deep eutectic solvent for the solid-phase extraction of protein. *Anal. Chim. Acta* 877 90–99. 10.1016/j.aca.2015.03.048 26002214

[B21] Jiang F. Huang S. Imadad K. Li C. (2012). Cloning and expression of a gene with phospholipase B activity from *Pseudomonas fluorescens* in *Escherichia coli*. *Bioreour. Technol.* 104 518–522. 10.1016/j.biortech.2011.09.112 22078969

[B22] Jiang X. Chang M. Wang X. Jin Q. Wang X. (2014). The effect of ultrasound on enzymatic degumming process of rapeseed oil by the use of phospholipase A1. *Ultrason. Sonochem.* 21 142–148. 10.1016/j.ultsonch.2013.07.018 24001661

[B23] Jiang X. F. Chang M. Wang X. Jin Q. Wang X. G. (2014). A comparative study of phospholipase A1 and phospholipase C on soybean oil degumming. *J. Am. Oil Chem. Soc.* 91 2125–2134. 10.1007/s11746-014-2555-6

[B24] Lamas D. L. Constenla D. T. Raab D. (2016). Effect of degumming process on physicochemical properties of sunflower oil. *Biocatal. Agric. Biotechnol.* 6 138–143. 10.1016/j.bcab.2016.03.007

[B25] Li F. Jiang X. Zhao J. Zhang S. (2015). Graphene oxide: a promising nanomaterial for energy and environmental applications. *Nano Energy* 16 488–515. 10.1016/j.nanoen.2015.07.014

[B26] Liu A. X. Yu X. W. Sha C. Xu Y. (2015). *Streptomyces violaceoruber* phospholipase A2: expression in *Pichia pastoris*, properties, and application in oil degumming. *Appl. Biochem. Biotechnol.* 175 3195–3206. 10.1007/s12010-015-1492-7 25618786

[B27] Manjula S. Jose A. Divakar S. Subramanian R. (2010). Degumming rice bran oil using phospholipase-A1. *Eur. Lipid J. Sci. Technol.* 113 658–664. 10.1002/ejlt.201000376 27904337

[B28] Martin S. F. Follows B. C. Hergenrother P. J. Trotter B. K. (2000). The choline binding site of phospholipase C (*Bacillus cereus*): insights into substrate specificity. *Biochemistry* 39 3410–3415. 10.1021/bi9919798 10727235

[B29] Nykter M. Kymäläinen H. R. Gates F. Sjöberg A. M. (2006). Quality characteristics of edible linseed oil. *Agric. Food Sci.* 15 402–413. 10.15171/apb.2018.013 29670845 PMC5896384

[B30] Pires L. N. Brandão G. C. Teixeira L. S. (2017). Determination of phospholipids in soybean lecithin samples via the phosphorus monoxide molecule by high-resolution continuum source graphite furnace molecular absorption spectrometry. *Food Chem.* 225 162–166. 10.1016/j.foodchem.2017.01.019 28193410

[B31] Qiu J. D. Peng H. P. Liang R. P. Xia H. (2010). Facile preparation of magnetic core-shell Fe3O4@Au nanoparticle/myoglobin biofilm for direct electrochemistry. *Biosens. Bioelectron.* 25 1447–1453. 10.1016/j.bios.2009.10.043 19942425

[B32] Qu Y. F. Sun L. X. Li X. Zhou S. Zhang Q. Sun L. B. (2016). Enzymatic degumming of soybean oil with magnetic immobilized phospholipase A(2). *LWT Food Sci. Technol.* 73 290–295. 10.1016/j.lwt.2016.06.026

[B33] Ravasi P. Braia M. Eberhardt F. Elena C. Cerminati S. Peiru S. (2015). High-level production of *Bacillus cereus* phospholipase C in *Corynebacterium glutamicum*. *J. Biotechnol.* 216 142–148. 10.1016/j.jbiotec.2015.10.018 26519562

[B34] Sampaio K. A. Zyaykina N. Wozniak B. Tsukamoto J. De Greyt W. Stevens C. V. (2015). Enzymatic degumming: degumming efficiency versus yield increase. *Eur. Lipid J. Sci. Technol.* 117 81–86. 10.1002/ejlt.201400218

[B35] Shao Y. Jing T. Tian J. Zheng Y. (2015). Graphene oxide-based Fe3O4 nanoparticles as a novel scaffold for the immobilization of porcine pancreatic lipase. *RSC Adv.* 5 103943–103955. 10.1039/C5RA19276E

[B36] Sheelu G. Kavitha G. Fadnavis N. W. (2008). Efficient immobilization of lecitase in gelatin hydrogel and degumming of rice bran oil using a spinning basket reactor. *J. Am. Oil Chem. Soc.* 85 739–748. 10.1007/s11746-008-1261-7

[B37] Stanley A. M. Treubrodt A. M. Chuawong P. Hendrickson T. L. Fleming K. G. (2007). Lipid chain selectivity by outer membrane phospholipase A. *Mol. J. Biol.* 366 461–468. 10.1016/j.jmb.2006.10.055 17174333

[B38] Su L. Q. Ji D. N. Tao X. M. Yu L. G. Wu J. Xia Y. M. (2017). Recombinant expression, characterization, and application of a phospholipase B from *Fusarium oxysporum*. *J. Biotechnol.* 242 92–100. 10.1016/j.jbiotec.2016.12.009 27940286

[B39] Sutto-Ortiz P. Camacho-Ruiz M. A. Kirchmayr M. R. Camacho-Ruiz R. M. Mateos-Díaz J. Noiriel A. (2017). Screening of phospholipase A activity and its production by new actinomycete strains cultivated by solid-state fermentation. *PeerJ* 5:e3524. 10.7717/peerj.3524 28695068 PMC5501967

[B40] Tolner B. Smith L. Begent R. Chester K. (2006). Production of recombinant protein in *Pichia pastoris* by fermentation. *Nat. Protoc.* 1 1006–1021. 10.1038/nprot.2006.126 17406338

[B41] Wang Y. Xuan Y. J. Zhang P. Jiang X. Ni Z. H. Tong L. J. (2009). Targeting expression of the catalytic domain of the kinase insert domain receptor (KDR) in the peroxisomes of *Pichia pastoris*. *FEMS Yeast Res.* 9 732–743. 10.1111/j.1567-1364.2009.00521.x 19473276

[B42] Wei T. Xu C. P. Yu X. Jia W. W. Yang K. P. Jia C. X. (2015). Characterization of a novel thermophilic phospholipase B from *Thermotoga lettingae* TMO: applicability in enzymatic degumming of vegetable oils. *J. Ind. Microbiol. Biotechnol.* 42 515–522. 10.1007/s10295-014-1580-7 25578305

[B43] Xie W. L. Huang M. Y. (2018). Immobilization of *Candida rugosa* lipase onto graphene oxide Fe3O4 nanocomposite: characterization and application for biodiesel production. *Energy Convers. Manag.* 159 42–53. 10.1016/j.enconman.2018.01.021

[B44] Yang M. Zheng C. Zhou Q. Huang F. H. Liu C. S. Wang H. (2013). Minor components and oxidative stability of cold-pressed oil from rapeseed cultivars in China. *J. Food Compost. Anal.* 29 1–9. 10.1016/j.jfca.2012.08.009

[B45] Yang P. Zhu X. Cao L. Cheng J. Zheng Z. Jiang S. (2017). Safety evaluation of *Bacillus cereus* isolated from smelly mandarin fish. *J. Food Meas. Charact.* 11 726–735. 10.1007/s11694-016-9442-9

[B46] Zhang L. Hellgren L. Xu X. B. (2007). Immobilization of phospholipase C for the production of ceramide from sphingomyelin hydrolysis. *J. Am. Oil Chem. Soc.* 84 237–247. 10.1007/s11746-006-1028-y

[B47] Zhuang W. He J. Zhu J. Zheng J. W. Liu X. J. Dong Y. H. (2016). Efficient nanobiocatalytic systems of nuclease P1 immobilized on PEG-NH2 modified graphene oxide: effects of interface property heterogeneity. *Colloids Surf. B Biointerfaces* 145 785–794. 10.1016/j.colsurfb.2016.05.074 27295495

[B48] Zufarov O. Schmidt S. Sekretar S. Cvengros J. (2009). Ethanoloamines used for degumming of rapeseed and sunflower oils as diesel fuels. *Eur. Lipid J. Sci. Technol.* 111 985–992. 10.1002/ejlt.200900025

